# Toll-like receptor 2 gene polymorphisms, pulmonary tuberculosis, and natural killer cell counts

**DOI:** 10.1186/1471-2350-11-17

**Published:** 2010-01-30

**Authors:** Yung-Che Chen, Chang-Chun Hsiao, Chung-Jen Chen, Chien-Hung Chin, Shih-Feng Liu, Chao-Chien Wu, Hock-Liew Eng, Tung-Ying Chao, Chia-Cheng Tsen, Yi-Hsi Wang, Meng-Chih Lin

**Affiliations:** 1 Division of Pulmonary and Critical Care Medicine, Department of Internal Medicine, Chang Gung Memorial Hospital-Kaohsiung Medical Center, Chang Gung University College of Medicine, Kaohsiung, Taiwan; 2 Graduate Institute of Clinical Medical Sciences, Chang Gung University Collage of Medicine, Kaohsiung, Taiwan; 3 Division of Rheumatology, Chang Gung Memorial Hospital-Kaohsiung Medical Center, Chang Gung University College of Medicine, Kaohsiung, Taiwan; 4 Department of Clinical Pathology, Chang Gung Memorial Hospital-Kaohsiung Medical Center, Chang Gung University College of Medicine, Kaohsiung, Taiwan

## Abstract

**Background:**

To investigate whether the toll-like receptor 2 polymorphisms could influence susceptibility to pulmonary TB, its phenotypes, and blood lymphocyte subsets.

**Methods:**

A total of 368 subjects, including 184 patients with pulmonary TB and 184 healthy controls, were examined for TLR2 polymorphisms over locus -100 (microsatellite guanine-thymine repeats), -16934 (T>A), -15607 (A>G), -196 to -174 (insertion>deletion), and 1350 (T>C). Eighty-six TB patients were examined to determine the peripheral blood lymphocyte subpopulations.

**Results:**

We newly identified an association between the haplotype [A-G-(insertion)-T] and susceptibility to pulmonary TB (p = 0.006, false discovery rate q = 0.072). TB patients with systemic symptoms had a lower -196 to -174 deletion/deletion genotype frequency than those without systemic symptoms (5.7% vs. 17.7%; p = 0.01). TB patients with the deletion/deletion genotype had higher blood NK cell counts than those carrying the insertion allele (526 vs. 243.5 cells/μl, p = 0.009). TB patients with pleuritis had a higher 1350 CC genotype frequency than those without pleuritis (12.5% vs. 2.1%; p = 0.004). TB patients with the 1350 CC genotype had higher blood NK cell counts than those carrying the T allele (641 vs. 250 cells/μl, p = 0.004). TB patients carrying homozygous short alleles for GT repeats had higher blood NK cell counts than those carrying one or no short allele (641 vs. 250 cells/μl, p = 0.004).

**Conclusions:**

TLR2 genetic polymorphisms influence susceptibility to pulmonary TB. TLR2 variants play a role in the development of TB phenotypes, probably by controlling the expansion of NK cells.

## Background

The innate immune system has evolved as the first line of defense against microorganisms, which involves specific pathogen recognition receptors such as toll-like receptors. It also plays a crucial role in initiating and directing the adaptive immune system[1]. Toll-like receptor 2 (TLR2) is capable of recognizing pathogen-associated molecular patterns expressed by *Mycobacterium tuberculosis *(Mtb), such as a 19-kDa lipoprotein, lipoarabinomannan, and soluble tuberculosis factor. This recognition leads to the production of inflammatory cytokines, such as tumor necrosis factor-α and interferon (IFN)-γ, that are predominantly secreted by T-helper-1 cells[2-5]. Increasing amounts of data suggest that genetic variants of TLR2 (GenBank accession number, NM_003264.3; MIM no. 603028) may play a role in determining the susceptibility to or severity of many infectious diseases[6].

The human TLR2 gene is located on chromosome 4q32 and is composed of 2 non-coding exons and 1 coding exon[7]. To date, more than 175 single-nucleotide polymorphisms (SNPs) or dinucleotide polymorphisms for the human TLR2 gene have been reported in the National Center for Biotechnology Information database http://www.ncbi.nlm.nih.gov. The G to A (Arg753Gln) polymorphism at position 2258 in exon 3 and the guanine-thymine (GT) microsatellite repeat polymorphism (100 bp upstream of the translational start site) in intron 2, have been associated with susceptibility to clinical tuberculosis (TB) disease in Turkish and Korean patients, respectively[8,9]. Another 2 polymorphisms within the TLR2 promoter region, namely, -16934 A>T and -196 to -174 insertion (Ins)>deletion (Del), have been associated with asthma and gastric cancer, respectively [10,11]. On the basis of the International HapMap Project, 2 tag SNPs (-15607 A>G and 1350 T>C) in the TLR2 region could be selected with a r^2 ^cutoff of 0.8 for the Han Chinese in Beijing using the algorithm-Tagger-pairwise Tagging[12,13]. Previous studies investigating the association between TLR2 polymorphisms and diseases have targeted individual genetic markers at a single locus[6,8-11,14]. An alternative approach is to use haplotype structures that are derived from allelic variants at a number of loci on a chromosome. Although synonymous SNPs in the coding region of the TLR2 gene have been associated with tuberculous meningitis in patients in Vietnam, there are no reports of the association between variants of this gene and the development of systemic symptoms of or pleural involvement in pulmonary TB[14].

Expression of TLR2 in the human immune system is most predominant in myelomonocytic cells, followed by B cells, CD56^+^16^+ ^cells, and T cells[15,16]. TLR2 is implicated in the activation of CD3-CD56^+ ^natural killer (NK) cells, which are a major source of early IFN-γ in tuberculous pleurisy[17]. It also directly controls the expansion and function of regulatory T cells and is involved in mediating B cell differentiation[18,19]. The association between TLR2 polymorphisms and lymphocyte subsets has not yet been determined.

We hypothesized that (1) TLR2 microsatellite polymorphism or SNPs may predispose Taiwanese people to pulmonary TB; (2) TLR2 gene polymorphisms may predispose patients with pulmonary TB to presenting with systemic symptoms or pleural involvement; and (3) TLR2 gene polymorphisms may influence blood lymphocyte subsets. The aim of the present study was to examine whether the genotypes defined by the 5 TLR2 gene polymorphisms located at -16934, -15607, -196 to -174, -100, and 1350 influence susceptibility to pulmonary TB, its clinical presentations, and peripheral blood lymphocyte subsets at diagnosis.

## Methods

### Study subjects

The study population consisted of 184 patients with newly diagnosed pulmonary TB, who were undergoing anti-TB treatment at the Pulmonary Department of the Chang Gung Memorial Hospital (Kaohsiung, Taiwan) during August 2006-July 2008. The specific criterion for enrollment was defined as the presence of at least 1 of the following: (1) clinical and radiological findings indicating pulmonary TB and at least 1 positive Mtb culture from 3 separate sputum examinations or 1 bronchial washing specimen obtained from bronchoscopy; (2) pathological evidence of TB on pleural or lung mass biopsy; and (3) clinical and radiological findings indicating improvement in suspected pulmonary TB with empirical anti-TB therapy. Patients with acquired immune deficiency syndrome or those receiving immunosuppressive agents were excluded. The control group consisted of 184 unrelated subjects recruited from the Center of Health Examination of Chang Gung Memorial Hospital (Kaohsiung, Taiwan). The specific criteria of enrollment were the absence of pulmonary lesions on chest radiographic examination and a negative history of TB disease. All the subjects of both the study and control groups are residents in Taiwan, where new TB cases per 100,000 populations were from 62.0 to 74.6 in the past seven years. We assume that people in Taiwan have similar exposure to M.tb, because the modes of its transmission are mainly through large droplets and small particle droplet nuclei. This study was approved by our institutional review board, and written informed consents were obtained from all subjects before blood sampling.

### Molecular techniques and genotyping

Genomic DNA was isolated from blood leukocytes using a genomic DNA purification kit (Puregene; Gentra systems, Minneapolis, Minnesota, USA). Genotyping was performed according to the methods described previously, with some modifications[9,11]. The nucleotide sequences of the primers used and the conditions for polymerase chain reactions (PCR) are listed in Table 1.

**Table 1:** Biological characteristics of the genotyped TLR2 polymorphisms and the primers and conditions used for PCR

Polymorphism (DNA position relative to ATG)	rs number	Primers and conditions for PCR
-100 (GT)n	rs34692294	Forward: ionhs5'-GCATTGCTGAATGTATCAGGGA-3' Reverse: 5'-CCACAAAGTATGTGCCATGGTCCAGTGCTTC-3' Condition: 95°C, 3 min; (95°C, 30 sec; 55°C, 30 sec; 72°C, 1 min) × 35 cycles; 60°C, 60 min
-16934 A>T	rs4696480	Forward: 5'-TGGTTCTGGAGTCTGGGAAG-3' Reverse: 5'-ACAGAACGGTCTCCAAGTAG-3' Condition: 94°C, 5 min; (94.1°C, 1 min; 59.3°C, 1 min; 72.2°C, 2 min)× 35 cycles; 72°C, 10 min.
-15607 A>G	rs1898830	Forward: 5'-GCAGCTGAAATCACAGAGCA Reverse: 5'-AGGATAATGGCCTCCTGCT Condition: 94°C, 5 min; (94°C,1 min; 67.1°C,40 sec; 72°C,2 min) × 30 cycle; 72°C, 10 min
-196 to -174 Ins>del	not available	Forward: 5'-cacggaggcagcgagaaa Reverse: 5'-ctgggccgtgcaaagaag Condition: 94°C, 5 min; (94°C,1 min; 64.5°C,1 min; 72°C,2 min) × 35 cycle; 72°C, 10 min
1350 T>C (S450S)	rs3804100	Forward: 5'-AACCGGAGAGACTTTGCTCA Reverse: 5'-AGTTATTGCCACCAGCTTCC Condition: 94°C, 5 min; (94°C,40 sec; 62°C,40 sec; 72°C,1 min) × 30 cycle; 72°C, 10 min

### Genotyping of the GT microsatellite repeat polymorphism by gene scan

PCRs with 5-carboxy-fluorescein (FAM)-labeled primers were carried out to amplify a region of about 250 bp flanking the GT microsatellite repeat region. The number of GT repeats was estimated by calculating the number of base pairs in the PCR products by using a sequencer (ABI Prism^®^3100 Genetic Analyzer; Applied Biosystems, USA) and Gene Scan analysis software.

### Genotyping of the -16934 A>T, -15607 A>G, 1350 T>C, and 2258 G>A polymorphisms by direct sequencing

Approximately 1 μg of sample DNA was added to a reaction mixture containing 2.5 μl 10 × buffer, 2 μl of each dNTP, 10 μmol of each primer, and 1.25 U of *Taq *DNA polymerase (Pro Taq Plus DNA polymerase). PCRs were carried out on a thermal cycler (GeneAmp^®^PCR system 9700; Applied Biosystem, Foster City, California, USA) under specific conditions and with primers to amplify regions of 1492, 618, 392, and 265 bp flanking the -16934 A>T, -15607 A>G, 1350 T>C, and 2258 G>A polymorphism loci, respectively. Genotyping was performed by sequence analysis of the PCR products using an ABI PRISM 3730 genetic analyzer (Applied Biosystems, Darmstady, Germany). We did not detect the 2258 G>A mutation in any subject in both the groups.

### Genotyping of the -196 to -174 insertion>deletion polymorphism by primer-specific PCR

The volume of the PCR reaction mixture was 25 μl, and the mixture contained 1 μg genomic DNA, 10 μmol of each primer, 2 μl of each dNTP, and 1.25 U of *Taq *DNA polymerase. The PCR products were visualized by electrophoresis on a 3.5% agarose gel and stained with ethidium bromide. A single band at 286 bp was judged to be the wild-type product; a single band at 264 bp, a homozygote variant; and 2 bands at 286 and 264 bp, a heterozygote variant.

### Determination of blood lymphocyte phenotypes by flow cytometry

To evaluate the expression of surface markers on freshly isolated peripheral blood mononuclear cells from 86 TB patients within 2 weeks of anti-TB treatment, we used fluorochrome-labeled monoclonal antibodies: anti-CD3-phycoerythrin (PE), CD4-fluorescein isothiocyanate (FITC), CD8-FITC, CD19-FITC, and CD56^+^16-FITC. All the antibodies were purchased from Beckman Coulter (Marseille, France). Acquisition was performed on a FACScalibur Flow Cytometer (Becton Dickinson, San Jose, California, USA), and 2 × 10^4 ^lymphocyte-gated events were collected according to their forward and side-scatter properties. These were further analyzed for the expression of CD3 and CD4 (or CD8, CD19, and CD56^+^16) in the FL1 and FL2 channels, respectively. The analysis of the data was performed using the SimulSET software. Absolute cell count was computed from the lymphocyte percentage of the differential white blood cell count.

### Statistical analysis

Deviation from the Hardy-Weinberg equilibrium was tested using a χ^2 ^goodness of fit test for each locus in each cohort. The global association between case-control status and each allele of GT repeat microsatellite polymorphism was tested using a likelihood ratio. The differences in allele frequencies and genotype distribution between the 2 groups were evaluated by a χ^2 ^test, and the odds ratios (OR) were calculated with a 95% confidence interval (CI). Pairwise linkage disequilibrium (LD) among the 4 non-microsatellite polymorphisms in the study population was measured by calculating the r^2 ^and D' statistics. LD blocks were defined on the basis of the internally developed solid spine method, which searches for a "spine" of strong LD running from one marker to another along the legs of the triangle in the LD chart, and the haplotype frequencies were estimated using the expectation-maximization algorithm with the Haploview software[12,13]. Haplotype counts for case-control association tests were obtained by summing the fractional likelihood of each individual for each haplotype. All tests were 2-tailed, and p < 0.05 was considered as significant. To assist in the interpretation of p-values given the number of statistical tests performed, false discovery rate q-values were calculated separately for single marker polymorphism and haplotype analyses. The *q *value estimates the proportion of results declared interesting that are actually false. A q value threshold of 0.2 was selected to separate false from true discoveries, so up to 20% of declared discoveries should be expected to be false [20,21].

The difference in the genotypic distribution between the TB phenotypes was evaluated in a dominant model by a χ^2 ^test in which the wild-type and heterozygote variant were compared with the homozygote variant, because the data fit the dominant model better than other models of inheritance, such as recessive and heterozygous advantage. Continuous variables between the 2 groups were analyzed by a Mann-Whitney *U*-test or independent T test, where appropriate.

## Results

### Demographics of the participants

Characteristics of cases and controls are listed in Table 2. The study population was all Asian in ethnicity. Age and male sex ratio were similar between the 2 groups. Traditional acquired risk factors, such as history of diabetes mellitus, malignancy, chronic bronchitis, and chronic renal insufficiency were more common in cases than in controls. Microbiological diagnosis was made in 142 (77.2%) TB patients; pathological diagnosis was made in 27 (14.7%), and clinical diagnosis was made in 15 (8.1%).

**Table 2:** Characteristics of Study Participants

Characteristic	TB Cases (n = 184)	Controls (n = 184)	P value
Age, mean ± standard deviation, years	56.7 ± 18.7	53.9 ± 11.5	0.082
Male, n (%)	133 (72.3)	122 (66.3)	0.214
Diabetes Mellitus, n (%)	37 (20.2)	11 (6)	< 0.001
Malignancy, n (%)	22 (12)	6 (3.3)	0.002
Chronic obstructive pulmonary disease, n (%)	21 (11.4)	9 (4.9)	0.022
Chronic renal failure, n (%)	9 (5.1)	2 (1.1)	0.026
Congestive heart failure, n (%)	1 (0.5)	5 (2.7)	0.1
Chronic hepatitis, n (%)	7 (3.8)	8 (4.3)	0.792

### Allele and genotype frequencies in TB patients and healthy controls

The genotype frequency distribution for all the 5 polymorphisms investigated was consistent with the Hardy-Weinberg equilibrium in the patients and control groups except for -16934A>T in the control cohort (p = 0.005). The allele frequencies of GT repeats between the 2 groups are summarized in Table 3. GT microsatellite polymorphism had no significant global association with risk of pulmonary TB (Likelihood Ratio 26.17, p = 0.052). When each allele was analyzed independently and the ones with minor allele frequency of < 5% was pooled together, no individual GT repeat alleles were associated with susceptibility to pulmonary TB. The overall distributions of short allele (S, number of GT repeats ≦ 16), middle allele (M, number of GT repeats = 17-22), and long allele (L, number of GT repeats ≧ 23) were not significantly different between the patients and control groups. No significant difference was observed individually between the patient and the control groups with respect to the allele or genotype frequency for the other four polymorphisms (Table 4).

**Table 3:** Allele frequencies of GT microsatellite repeat dinucleotides polymorphism in cases and control subjects

Allele	Cases n (%)	Controls n (%)	OR (95% CI)	P value	FDR q value
GT11-12	20 (5.4)	25 (6.8)	0.79 (0.43-1.45)	0.442	0.636
GT13	65 (17.7)	70 (19)	0.91 (0.63-1.33)	0.634	0.749
GT14-18	7 (1.9)	4 (1.1)	1.77 (0.51-6.08)	0.362	0.588
GT19	26 (7.1)	16 (4.3)	1.67 (0.88-3.17)	0.112	0.485
GT20	81 (22	87 (22.8)	0.95 (0.68-1.35)	0.791	0.857
GT21-22	23 (6.3)	30 (8.2)	0.75 (0.43-1.32)	0.318	0.588
GT23	48 (13)	61 (16.6)	0.76 (0.5-1.14)	0.177	0.588
GT24	73 (19.8)	55 (15)	1.4 (0.95-2.06)	0.086	0.485
GT25	13 (3.5)	21 (5.7)	0.61 (0.3-1.23)	0.16	0.52
GT26-27	7 (1.9)	1 (0.3)	7.12 (0.87-58.13)	0.033	0.429
S	88 (23.9)	100 (27.2)	0.84 (0.6-1.17)	0.31	0.588
M	136 (37)	127 (34.5)	1.11 (0.82-1.5)	0.489	0.636
L	143. (38.9)	141 (38.3)	1.02 (0.76-1.38)	0.88	0.88

OR = Odds ratio; CI = confidence interval; FDR = false discovery rate

**Table 4:** Genotype and allele frequencies of TLR 2 gene polymorphisms in TB patients and control subjects*

Polymorphism	TB patients, N = 184	Control subjects, N = 184	OR (95% CI)	P value
	N (%)	N (%)		
-16934 A>T				
AA	64 (34.8)	71 (38.6)		0.571
TA	83 (45.1)	73 (39.7)		
TT	37 (20.1)	40 (21.7)		
A	211 (57.3)	215 (58.4)		
T	157 (42.7)	153 (41.6)	1.05 (0.78-1.4)	0.765
-15607 A>G				
AA	48 (26.1)	58 (31.5)		0.481
AG	101 (54.9)	91 (49.5)		
GG	35 (19)	35 (19)		
A	197 (53.5)	207 (55.5)		
G	171 (46.5)	161 (44.5)	1.08 (0.81-1.45)	0.592
-196 to -174 Ins>Del				
Ins/Ins	93 (50.5)	91 (49.5)		0.974
Ins/Del	71 (38.6)	72 (39.1)		
Del/Del	20 (10.9)	21 (11.4)		
Ins	257(69.8)	254 (69)		
Del	111 (30.2)	114 (31)	0.96 (0.7-1.32)	0.81
1350 T>C				
TT	131 (71.2)	121 (65.8)		0.497
TC	45 (24.5)	55 (29.9)		
CC	8 (4.3)	8 (4.3)		
T	307 (83.4)	297 (80.7)		
C	61 (16.6)	71 (19.3)	0.83 (0.57-1.21)	0.337

* All p values for Hardy-Weinberg equilibrium in each cohort were > 0.05 except for the one of polymorphism b (-16934A/T) in control cohort (p = 0.005)

### Association of TLR2 haplotype with pulmonary TB

**Figure 1:**
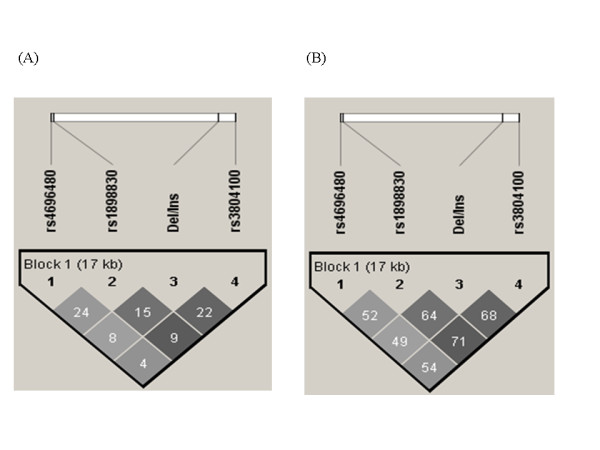
**Linkage disequilibrium plots**. TLR2 gene loci of the four investigated polymorphisms on chromosome 4q32, and description of intra-genetic linkage disequilibrium patterns: (A) and (B) r^2 ^and D' plots, respectively.

Figure 1 shows a graphical representation of LD between the loci of the four non-microsatellite polymorphisms. The physical distance between polymorphisms -16934 A>T and 1350 T>C on chromosome 4 is approximately 17 kb. Moderate LD was observed among the four polymorphisms (D' > 0.5). Using the 4 non-microsatellite polymorphisms, haplotype frequencies were estimated by the Haploview software. Haplotype [A-G-(Ins)-T] was associated with susceptibility to pulmonary TB (OR, 1.99; 95% CI, 1.21-3.25; p = 0.006, q = 0.072) (Table 5).

**Table 5:** Estimation of TLR2 haplotype frequencies in the study population by using the expectation-maximization algorithm with the Haploview software

Haplotype	TB patients, N = 184	Control subjects N = 184	OR (95% CI)	P value	FDR q value
	Counts ratios (frequency %)	Counts ratios (frequency %)			
T-G-(Ins)-T	97.3/270.7 (26.4)	113.9/254.1 (31)	0.8 (0.58-1.1)	0.166	0.573
A-A-(Ins)-T	66.4/301.6 (18)	75.1/292.9 (20.4)	0.85 (0.59-1.23)	0.399	0.573
A-A-(Del)-C	37.1/330.9 (10.1)	46.6/321.4 (12.7)	0.76 (0.48-1.21)	0.246	0.573
A-A-(Del)-T	38.8/329.2 (10.5)	42/326 (11.4)	0.92 (0.58-1.46)	0.724	0.714
A-G-(Ins)-T	50.1/317.9 (13.6)	27.2/340.8 (7.4)	1.99 (1.21-3.25)	0.006	0.072
T-A-(Ins)-T	27.7/340.3 (7.5)	21.4/346.6 (6.3)	1.36 (0.76-2.44)	0.301	0.573
Other**	7.0/361 (1.9)	5.9/362.1 (1.6)	1.17 (0.39-3.52)	0.78	0.714

OR = Odds ratio; CI = confidence interval; FDR = false discovery rate

* Haplotypes consisting of 4 alleles at -16934A>T, --15607A>G, -196 to -174 insertion>deletion, and 1350T>C (polymorphism b, c, d, e)

** Combined rare haplotypes

### Associations between the -196 to -174 Del/Del and 1350 CC genotypes with TB phenotypes

When TB patients were divided into those with (105/184) or without (79/184) systemic symptoms, including fever, weight loss, or anorexia, the only significant difference between the 2 subgroups was with respect to the -196 to -174 Ins>Del polymorphism. TB patients with systemic symptoms had a significantly lower Del/Del genotype frequency than those without systemic symptoms (5.7% vs. 17.7%; OR, 0.28; 95% CI, 0.1-0.77; p = 0.01). When the patients with pulmonary TB were divided into those with (40/184) or without (144/184) pleural involvement, defined as the presence of pleural effusion on chest X-ray (CXR), the only significant difference between the 2 subgroups was with respect to the 1350 T to C SNP. TB patients with pleural effusions had a significantly higher 1350 CC genotype frequency than those without pleural effusions (12.5% vs. 2.1%; OR, 6.71; 95% CI, 1.53-29.45; p = 0.004) (Table 6.).

**Table 6:** Association of TLR2 -196 to -174 deletion/deletion and 1350 CC genotypes with TB phenotypes.

TB phenotype	Polymorphism -196 to -174 Ins>Del		OR (95% CI)	P value
	Ins/Ins + Ins/Del	Del/Del		
Systemic symptoms				
Yes, n = 105	99 (94.3)	6 (5.7)	0.28 (0.1-0.77)	0.01
No, n = 79	65 (82.3)	14 (17.7)		
				
	Polymorphism 1350 T>C			
	TT + TC	CC		
Pleural involvement				
Yes, n = 40	35 (87.5)	5 (12.5)	6.17 (1.53-29.45)	0.004
No, n = 144	141 (97.9)	3 (2.1)		

Odds ratio (OR) and 95% confidence interval (CI) are reported when the common allele (insertion or T) is dominant.

### Associations between the TLR2 genotypes and blood absolute NK cell counts in TB patients

**Figure 2:**
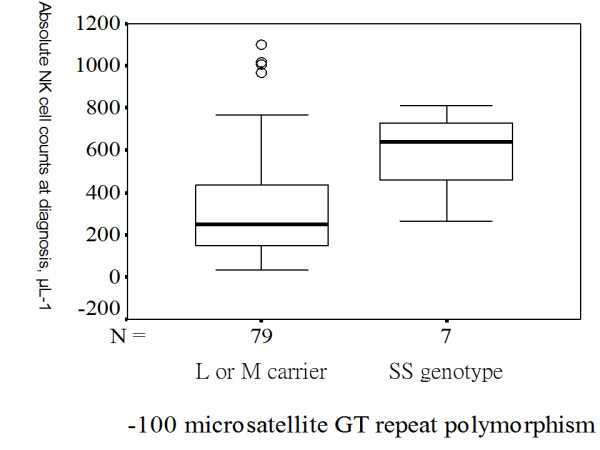
**Homozygous TLR2 -100 GT repeat polymorphism and absolute natural killer (NK) cell counts measured at diagnosis**. TB patients carrying homozygous S alleles for TLR2 -100 microsatellite GT repeat polymorphism (SS genotype) had higher blood absolute NK cell counts compared with those carrying one S allele or without carrying S allele (p = 0.004). The box plots show 25^th^, 50^th^, and 75^th ^percentiles, maximal, minimal, outliers (○).

**Figure 3:**
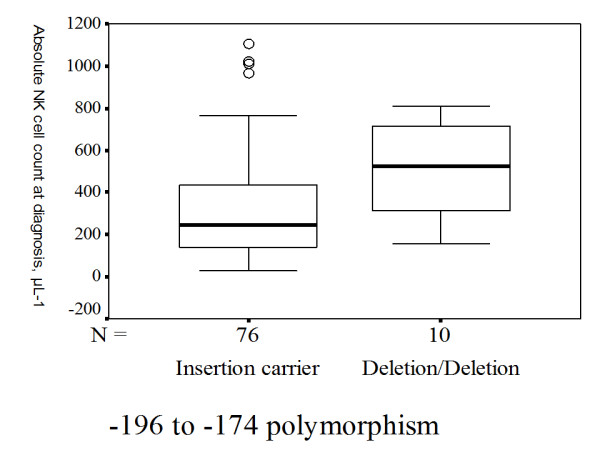
**Homozygous TLR2 -196 to -174 Ins>Del polymorphism and absolute natural killer (NK) cell counts measured at diagnosis**. TB patients carrying homozygous rare alleles for TLR2 -196 to -174 deletion/deletion genotype had higher blood absolute NK cell counts compared with those carrying common insertion allele (p = 0.009). The box plots show 25^th^, 50^th^, and 75^th ^percentiles, maximal, minimal, outliers (○).

**Figure 4:**
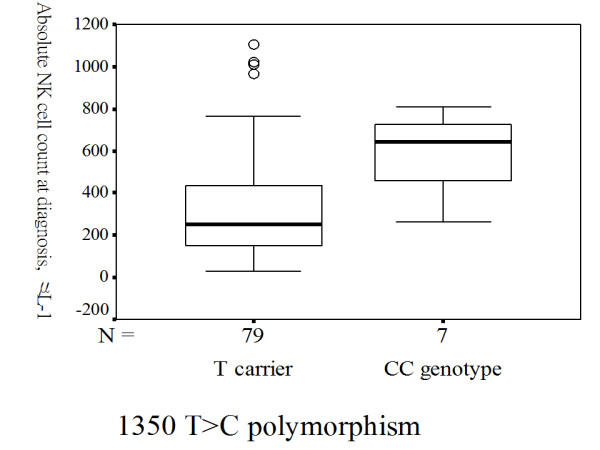
**Homozygous TLR2 1350 T>C polymorphism and absolute natural killer (NK) cell counts measured at diagnosis**. TB patients carrying homozygous rare alleles for TLR2 1350 CC genotype had higher blood absolute NK cell counts compared with those carrying common allele (p = 0.004). The box plots show 25^th^, 50^th^, and 75^th ^percentiles, maximal, minimal, outliers (○).

TB patients carrying homozygous S alleles had higher blood absolute NK cell counts compared with those carrying one S allele or those without carrying S allele [641 (419-743) vs. 250 (149-440), p = 0.004] (Figure 2). TB patients with the del/del homozygote genotype had a significantly higher blood absolute NK cell counts calculated at diagnosis than those carrying the common insertion allele [526 (301-721.3) vs. 243.5 (137.8-438) cells/μl; p = 0.009] (Figure 3). TB patients with the 1350 CC homozygote variant had a significantly higher blood absolute NK cell counts at diagnosis than those carrying the common T allele [641 (419-743) vs. 250 (149-440) cells/μl; p = 0.004] (Figure 4). In contrast, no significant effect of any of the 5 TLR2 polymorphisms was observed on other lymphocyte subsets, including CD19^+ ^B cells, CD4^+ ^T cells, and CD8^+ ^T cells.

## Discussion

In this genetic analysis of the TLR2 polymorphisms, we newly identified an association between the specific haplotype [A-G-(Ins)-T] and susceptibility to pulmonary TB in the Taiwanese population. Of the 4 non-microsatellite polymorphisms investigated, none had an effect individually on susceptibility to pulmonary TB. We demonstrated a distinct role of TLR2 polymorphisms on the development of different TB phenotypes. This is the first report stating that TLR2 polymorphisms were associated with elevated blood absolute NK cell counts.

Previous studies showed that Genotypes with shorter GT repeats were more common among Korean patients with pulmonary TB and non-tuberculous mycobacterial lung disease[9,22]. Our data showed that neither individual GT repeat allele nor the short repeat (S) was associated with susceptibility to TB. This indicates that the microsatellite marker may not be the functional disease-causing allele or a marker of other unknown causative mutation. Functional studies on the polymorphic (GT)n repeat have shown inconsistent results. One study reported that either the shortest [GT)n = 12] or longest [(GT)n = 28] alleles, rather than middle [(GT)n = 20] could lead to higher promoter activity when exposed to external stimuli[9]. The other study showed that shorter [(GT)13] allele had lower promoter activity than middle [(GT)20] and longer [(GT)24] alleles[23]. An association between SS genotype and elevated NK cell counts was observed in our study, and indicate that TLR2 genetic variant may play a role in controlling the expansion of NK cells. Further study is needed to clarify the role of microsatellite GT repeat in mediating TLR2 transcription activity or the expansion of lymphocyte subsets.

Haplotypes represent the majority of common variations in a gene because the human genome is organized into haplotype blocks, which are undisrupted by recombination during population history of gene[12]. The specific haplotype [A-G-(Ins)-T] consisting of 1 rare allele at -15607 position and 3 common alleles at other loci showed a significant association with susceptibility to pulmonary TB. In the European population, the -16934 A>T SNP has been reported with allele A being present in an equal frequency to allele T[10]. However, in the Taiwanese population, -16934 A is a common allele occurring at a frequency of 57.3-58.4%. This may lead to the differences in the association between these polymorphisms and the disease in different populations. Of the 5 polymorphisms that were investigated, 2 have been reported to have an effect on TLR2 gene expression. The -196 to -174 deletion allele in the 5' un-translated region and the short GT repeat allele at intron2 tended to have lower promoter activity than that in the wild-type allele[23,24]. On the basis of the FASTSNP analysis, the 1350 T>C variant, a synonymous SNP located at the coding region of exon 3, has been predicted to have a functional effect on diminishing the number of the putative exonic splicing enhancer motifs[25]. The findings of these functional studies provided a possible explanation for why the specific haplotype might be linked to pulmonary TB and why the specific genotype might be related to TB phenotypes or blood lymphocyte subsets. Further studies are required to clarify the functional effect of the -15607 A>G SNP.

In a recent study, systemic symptoms were reported to be absent in 25% of TB patients, with fever and weight loss being absent in 37% and 38% patients, respectively[26]. On the basis of our study, the -196 to -174 del/del homozygote genotype might have a preventive effect on the development of systemic symptoms, including fever, anorexia, and weight loss. An association between the -196 to -174 Del/Del genotype and steroid-dependent ulcerative colitis has been recently reported, although the functional significance of this association was not explored[27]. We evaluated the association between peripheral blood lymphocyte subpopulations and the -196 to -174 genetic variant in TB patients, and found higher blood absolute NK cell counts in those patients with the del/del genotype. Human NK cells have been demonstrated to directly recognize Mycobacterium via TLR2, and release TNF-α and IFN-γ[28]. Compartmentalization of the CD4(+) T lymphocytes in the infected lungs with a reciprocal decrease in peripheral blood counts of the same lymphocyte subset has been demonstrated in patients with higher grades of pulmonary TB[29]. Thus, we speculated that decreased counts of NK cells are recruited to the TB lesions in the lung parenchyma in patients with higher blood NK cell counts and, hence, the levels of pro-inflammatory cytokines released from these cells would be lower in such patients with a lower promoter activity of the TLR2 gene. This indicates that patients with the homozygote -196 to -174 del/del genotype may possess innate immune mechanisms of resistance to the development of systemic symptoms, which may be attributed to the decreased levels of cytokines such as TNF-α and IFN-γ.

The frequency of pleural involvement in TB has been reported to vary from 4% to 23% in different populations[30]. We observed an association between the 1350 CC genotype and the presence of pleural effusions in patients with pulmonary TB. In addition, patients with the CC homozygote variant had significantly higher blood absolute NK cell counts than those carrying the common T allele. Published data have demonstrated that Mtb-induced IFN-γ production by NK cells requires cross talk with antigen-presenting cells via TLR2, and that TLR2 expression of NK cells within pleural fluid is down-regulated compared with that in peripheral blood in TB pleurisy[16,17]. Our data suggest that TLR2-genetically determined high NK cell counts are likely to predispose TB patients to pleural involvement. However, the reason behind the altered NK cell counts associated with both the 1350 CC and -196 to -174 Del/Del genotypes needs to be further analyzed. By comparing the NK cell counts between patients with different genotypes, we identified 4 outlier values in patients carrying the common allele. This indicates that genetic variants of other immunological mediators may also contribute to controlling the expansion of NK cells.

The statistical power to detect significant associations with rare genetic variants was limited by sample size. Based on the sample size, we estimated that for a haplotype with a prevalence of 10%, there was 86.6% power to detect a 50% change in risk. We also estimated the power to be 74.4% for the comparison of NK cell counts between patients with the Insertion carrier and Del/Del genotype, and 92.3% between patients with the 1350 T carrier and CC genotype, using the standard *t *test formulations with a simple adjustment to the sample sizes in our study and an α error of 0.05 with PASS 2005 (NCSS, Kaysville, Utah, USA) software. The population of individuals with homozygous genetic variant was too small in our study to draw strong conclusions about risk differences by individual TLR2 genetic variant. This is a major limitation of this study, and there is a need to replicate and validate these association results in another large cohort. On the other hand, genetic factors represent only part of the risk associated with complex disease phenotypes, and multiple genetic products combine to produce a phenotype. Thus, a minor effect of individual genetic variant is more frequently observed in complex diseases. Another limitation of this study is lack of adjustments for interactions with the acquired risk factors, such as DM. However, these do not generally confound genetic associations except through selection bias or modification of the TLR2 gene-pulmonary TB association.

## Conclusions

We observed an association between the specific TLR2 haplotype and susceptibility to pulmonary TB. In patients with pulmonary TB, both the -196 to -174 Del/Del and 1350 CC genotypes were associated with an increased blood absolute NK cell counts and might have an influence on the development of systemic symptoms or pleural involvement, respectively.

## Abbreviations

TLR2: Toll-like receptor 2; SNPs: single-nucleotide polymorphisms; GT: guanine-thymine; TB: tuberculosis; IFN: interferon; Mtb: *Mycobacterium tuberculosis*; OR: odds ratios; CI: confidence interval; NK: natural killer; PCR: polymerase chain reactions; LD: linkage disequilibrium.

## Competing interests

The authors declare that they have no competing interests.

## Authors' contributions

YC performed the genotyping, carried out the statistical analysis, and drafted the manuscript. ML and CH interpreted and analyzed the data, and critically revised and approved the manuscript. CJC elaborated the design of the study. HE performed the flowcytometric analysis. CHC, SL, CW, TC, YW, and CT recruited the study subjects, reviewed the chart, and collected the samples. All authors read and approved the final manuscript.

## Pre-publication history

The pre-publication history for this paper can be accessed here:

http://www.biomedcentral.com/1471-2350/11/17/prepub
